# The Following List of Deaths Is Given by the Bills of Mortality for the Last Three Months

**Published:** 1799-11

**Authors:** 


*The following List of Deaths is given by the Bills of Mortality for the last Three Months.*

| | | | |
|---|---|---|---|
| Abscess | 4 | French Pox | 3 |
| Abortive | 7 | Gout | 16 |
| Aged | 226 | Hooping Cough | 47 |
| Ague | 1 | Jaundice | 22 |
| Apoplexy | 16 | Inflammation | 99 |
| Asthma | 70 | Lunatic | 23 |
| Cancer | 8 | Measles | 51 |
| Childbed | 22 | Mortification | 55 |
| Colic | 3 | Palsy | 35 |
| Consumption | 972 | Pleurisy | 2 |
| Convulsions | 785 | Rupture | 5 |
| Croup | 2 | Small-pox | 161 |
| Dropsy | 217 | Still born | 91 |
| Evil | 2 | Suddenly | 31 |
| Fever | 334 | Teething | 44 |
| Flux | 3 | Water in the head | 21 |

---

The diseases taken notice of in the last report still continue to prevail. Disorders of the bowels are very frequent, and, in some instances, obstinate. In most of the cases they constitute the principal disease; but in some few, they blend themselves with other diseases of the system. In addition to the diarrhœa and dysentery, which were mentioned before, some cases of cholera morbus have occurred. Several persons in one family were affected by it at the same time; the symptoms, however, were mild, and a recovery was soon obtained.

The hooping cough and measles have prevailed amongst children; but have not in general been attended with any formidable symptoms. The number of instances in which the former of these diseases has proved fatal, as stated in the list of deaths given by the Bills of Mortality for the last three months, as annexed to this Report, forms a pleasing comparison with the account of the same period in the last year. In the months of July, August, and September, 1798, 104 fell a sacrifice to this disease. In the same months of the present year only 47 died.

